# SEEKING SUPPORT FOR MENTAL HEALTH IN EAST ASIAN AMERICAN DEMENTIA CAREGIVERS: A GROUNDED THEORY STUDY

**DOI:** 10.1093/geroni/igae098.3381

**Published:** 2024-12-31

**Authors:** Eunjung Ko, Judith Tate, Kathy Wright, Karen Rose

**Affiliations:** New York University, New York, New York, United States; The Ohio State University, Columbus, Ohio, United States; The Ohio State University, Columbus, Ohio, United States; The Ohio State University, Columbus, Ohio, United States

## Abstract

Asian Americans in general are reluctant to seek mental health support. This can be detrimental to those who are also dementia caregivers. Unfortunately, Asian Americans and each subgroup in this population are underrepresented in dementia caregiving research. We explored help-seeking behaviors for mental health in East Asian American dementia caregivers using a qualitative analysis based on constructivist grounded theory. We conducted 60–90 minute interviews with each participant. Interviews were recorded and transcribed verbatim. To date, 16 participants have completed the interviews using purposive sampling. Thirteen were females, including 12 daughters and one daughter-in-law. Twelve were first-generation immigrants. The average caregiving duration was 40 months. We developed seven themes—“providing care,” “individual values,” “a need for support,” “seeking support,” “barriers to using support,” “benefits from support,” “living now and the future”—comprising 18 categories. While providing care, caregivers observed care recipient’s symptoms, experienced caregiving challenges, and encountered personal life events, leading them to need and seek help. Individual values of caregivers—cultural acceptance, faith, caregiving competence, dyadic connectedness—could influence how they perceive their situations. Caregivers then sought formal, informal, and self-support separately or in combination, albeit with several barriers to accessing support. Through the support, caregivers could achieve benefits (connecting, learning, relieving, reassuring). The benefits, along with individual values while providing care, could affect caregivers’ viewpoints on the present and future. These initial findings can provide researchers with valuable insights into the imperative need for focused research on Asian American communities and the development of strategies to bolster mental health support for this population.

